# Manipulation of visual biofeedback during gait with a time delayed adaptive Virtual Mirror Box

**DOI:** 10.1186/1743-0003-11-101

**Published:** 2014-06-10

**Authors:** Gabor J Barton, Alan R De Asha, Edwin CP van Loon, Thomas Geijtenbeek, Mark A Robinson

**Affiliations:** 1 Research Institute for Sport and Exercise Sciences, Liverpool John Moores University, Tom Reilly Building, Byrom Street, Liverpool L3 3AF, United Kingdom; 2 School of Engineering, Design & Technology, University of Bradford, Bradford, United Kingdom; 3 Motek Medical B.V., Amsterdam, The Netherlands

**Keywords:** Virtual reality, Mirror box, Rehabilitation, Gait, Amputees, Phantom limb pain, Stroke, Cerebral palsy, Complex regional pain syndrome

## Abstract

**Background:**

A mirror placed in the mid-sagittal plane of the body has been used to reduce phantom limb pain and improve movement function in medical conditions characterised by asymmetrical movement control. The mirrored illusion of unimpaired limb movement during gait might enhance the effect, but a physical mirror is only capable of showing parallel movement of limbs in real time typically while sitting. We aimed to overcome the limitations of physical mirrors by developing and evaluating a Virtual Mirror Box which delays the mirrored image of limbs during gait to ensure temporal congruency with the impaired physical limb.

**Methods:**

An application was developed in the CAREN system’s D-Flow software which mirrors selected limbs recorded by real-time motion capture to the contralateral side. To achieve phase shifted movement of limbs during gait, the mirrored virtual limbs are also delayed by a continuously calculated amount derived from past gait events. In order to accommodate non-normal proportions and offsets of pathological gait, the movements are morphed so that the physical and virtual contact events match on the mirrored side. Our method was tested with a trans-femoral amputee walking on a treadmill using his artificial limb. Joint angles of the elbow and knee were compared between the intact and mirrored side using cross correlation, root mean squared difference and correlation coefficients.

**Results:**

The time delayed adaptive virtual mirror box produced a symmetrical looking gait of the avatar coupled with a reduction of the difference between the intact and virtual knee and elbow angles (10.86° and 5.34° reduced to 4.99° and 2.54° respectively). Dynamic morphing of the delay caused a non-significant change of toe-off events when compared to delaying by 50% of the previous gait cycle, as opposed to the initial contact events which showed a practically negligible but statistically significant increase (p < 0.05).

**Conclusions:**

Adding an adaptive time delay to the Virtual Mirror Box has extended its use to treadmill gait, for the first time. Dynamic morphing resulted in a compromise between mirrored movement of the intact side and gait events of the virtual limbs matched with physical events of the impaired side. Asymmetrical but repeatable gait is expected to provide even more faithful mirroring.

## Background

Visual illusions provided by a mirror box can reduce phantom limb pain (PLP) experienced by amputees [1]. These illusions are created by placing a mirror in the mid-sagittal plane of the person so that the amputated limb appears intact. Regular exposure to such illusions is termed ”Mirror Therapy”, and when used repeatedly, it affords continuous pain relief in a substantial percentage of PLP sufferers [2-5]. The benefits of mirror therapy are not exclusive to PLP, it has also been used for motor rehabilitation of stroke [6,7], complex regional pain syndrome (CRPS) [8] and cerebral palsy [9], although the successful use of mirror therapy in conditions other than PLP is mainly evidenced in small sample case studies [8]. Despite this, mirror therapy shows considerable promise to the extent that the presence of a physical mirror may not actually be necessary as similar effects have been shown using motor imagery [5] and manipulated video footage [10]. These examples strongly point to a significant role for the visual system in chronic pain and in motor rehabilitation, all of which can be exploited with virtual reality (VR).

Mirror therapy has been provided using virtual reality in a number of different ways. In sitting upper limb amputees, reflected digital video projected onto a screen above the missing limb successfully decreased PLP following training twice a week for eight weeks. Reduction of pain was maintained four weeks after the intervention [11]. An immersive VR protocol was developed to provide mirror therapy [12]. This protocol overcame a weakness of traditional mirror therapy in that it did not require a mirror to be kept in the body’s mid-sagittal plane, or to keep the head turned towards the mirror. All of these studies indicate that if the visual stimulus is sufficiently powerful, the use of VR mirroring for chronic pain and motor rehabilitation is worth investigating.

Whilst all of the aforementioned studies have shown considerable potential for pain reduction and motor rehabilitation, they share the weakness that using either a physical mirror or a fully immersive virtual environment restricts the choice of tasks due to the participants’ seated position. A physical mirror allows only spatial mirroring and so this prohibits phase shifted or parallel reaching type movements [13] which would require a temporal delay. Furthermore, open kinetic chain symmetrical limb movements while sitting with the limbs in the air have limited real-life equivalents and therefore lack ecological validity. Importantly, chronic pain occurs not only in static but also during dynamic activities, in fact exercise may even be used as a coping strategy [14].

The extension of mirror therapy to activities such as walking gait has been avoided because performing such functional activities is not feasible within the constraints of a physical mirror box or fully immersive VR. The mirroring of limbs during walking would give beneficial real-time visual feedback to patients not only with chronic pain e.g. PLP, CRPS but also those with motor impairment e.g. stroke or cerebral palsy during a functional activity of daily living. Through techniques such as real-time three-dimensional motion capture and partially immersive VR, motor impairment can be assessed and mirror therapy administered to benefit such populations during dynamic activities. The aim of this study was to develop a Virtual Mirror Box application for gait by overcoming the limitations of the conventional mirror box.

## Methods

**Figure 1:**
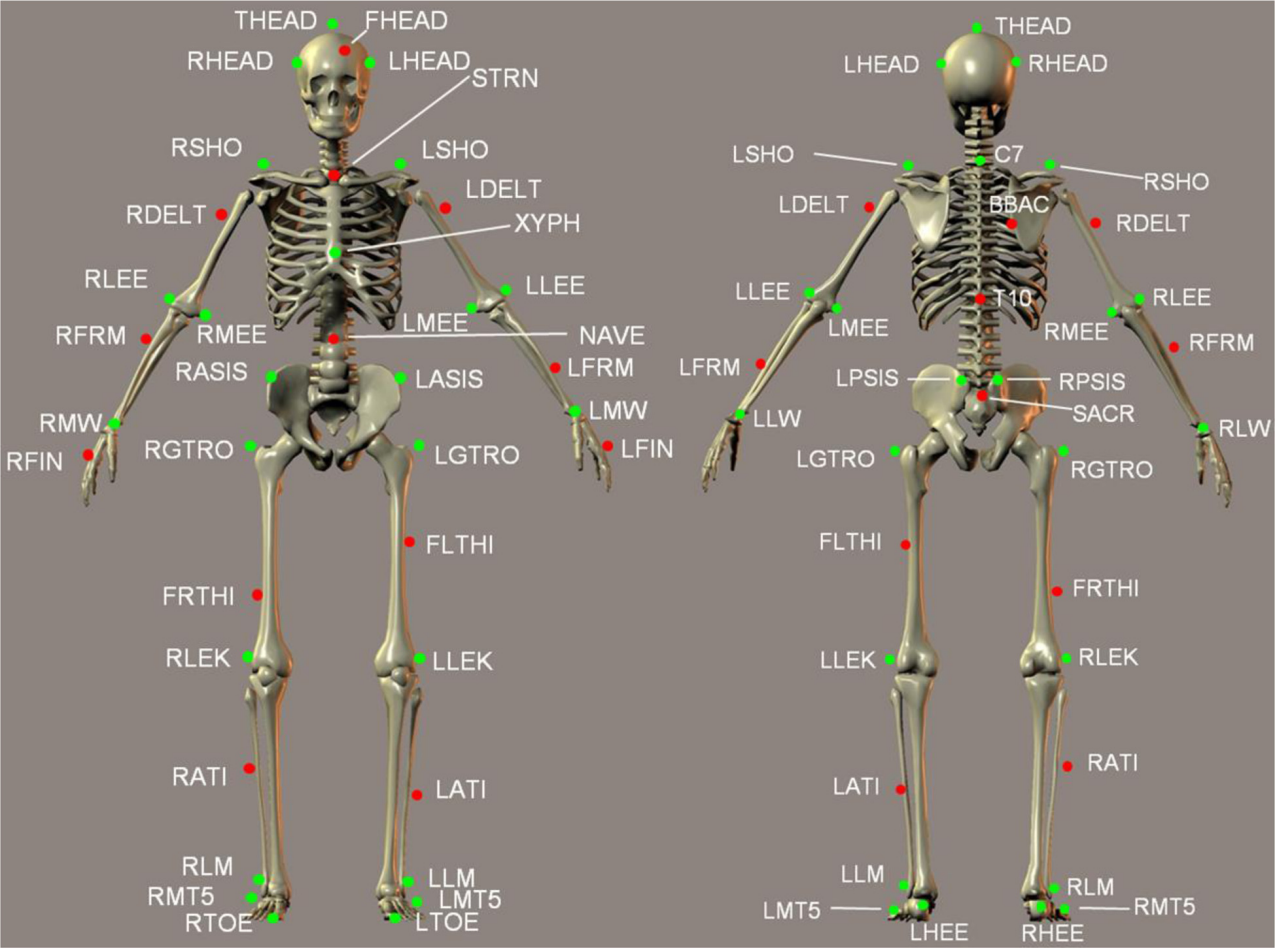
**The Human Body Model is defined by 47 surface markers attached over bony landmarks, for details see **[15]**.**

**Figure 2:**
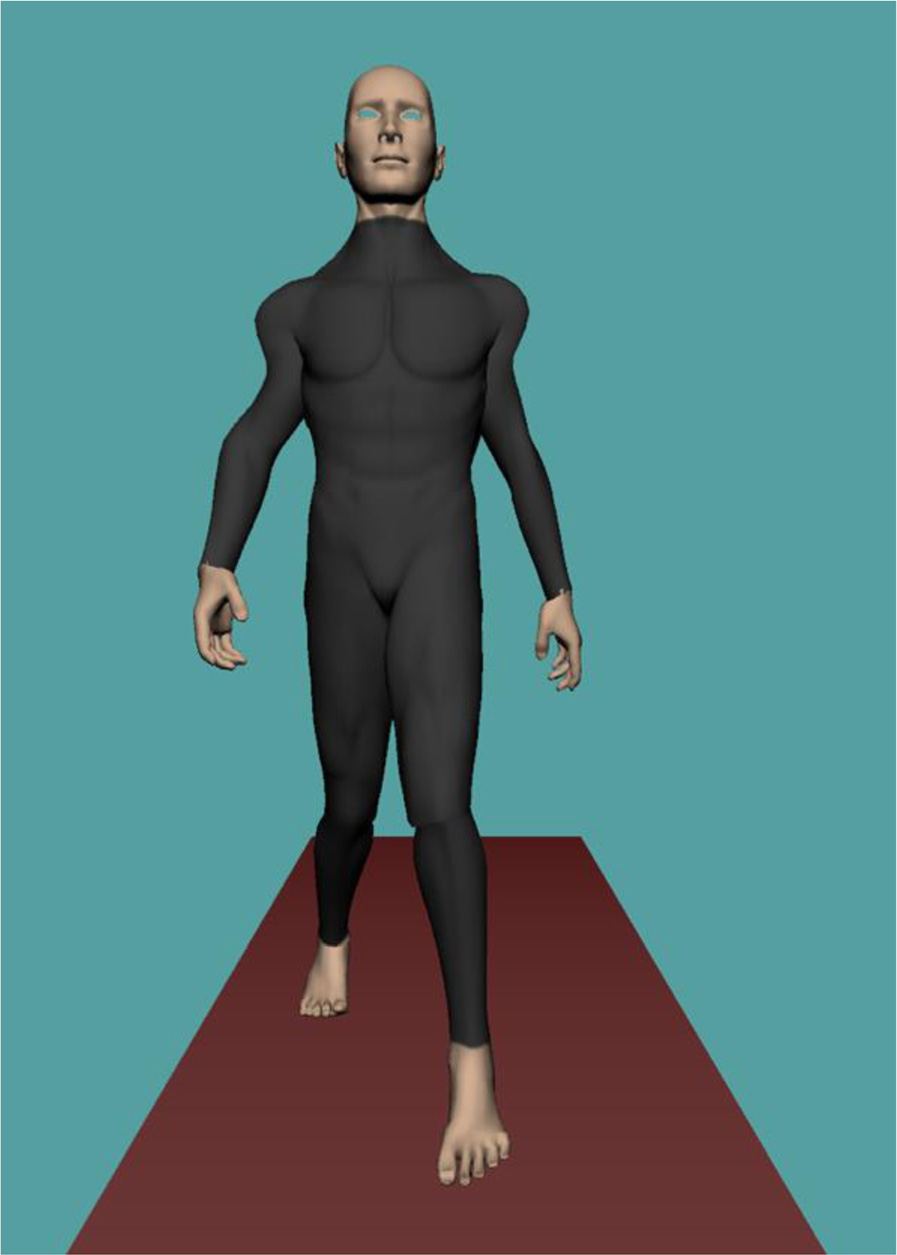
The view seen by the patient on a display in front of the treadmill.

Real-time visualisation of a person’s whole body movement during treadmill gait can be achieved with the Computer Assisted Rehabilitation Environment (CAREN system, Motek Medical, Amsterdam, Netherlands). Reflective markers (15 mm) are attached to the body over anatomical landmarks according to the Human Body Model (HBM [15]) (Figure 1). The three dimensional positions of 47 markers are reconstructed using 16 Vicon MX cameras (Vicon, Oxford, UK) sampling at 100 Hz and Nexus 1.8.2 software, which labels and streams data in real-time through a local area network to a separate computer which runs the D-Flow software (Motek Medical). An avatar wearing a tight fitting black whole body suit is visualised on a display which can act as a virtual mirror facing the participant to provide visual feedback of gait while walking on a treadmill (Figure 2). Age and gender specific avatars can be created even using photos of the participant.

In order to mirror one side of the body to the other side, a second type of virtual mirror is necessary. The custom made Virtual Mirror Box (VMB) within D-Flow’s MoCap module [16] mirrors the arm and/or leg of a selected side in place of the other side using a time delay as input. Feeding zero delay to VMB replicates the functionality of a physical mirror box with the added possibility of positioning the avatar anywhere by moving the display. In order to produce time delayed mirroring, an application created in D-Flow by linking various processing modules calculates initial contact and toe-off events of both sides in order to determine the time delay which is applied to the mirrored arm and/or leg during gait. The simultaneously applied spatial mirroring and temporal delay ensures that movement of the virtual mirrored limb matches movement of the impaired physical limb as closely as possible. Such spatial and temporal congruence between the virtual and physical limb is necessary for the patient to develop a sense of ownership of the virtual limbs.

### Technical challenges and solutions

#### ***Detection of gait events***

The time delay required for mirroring limbs during gait is essentially the phase shift between movement of the right and left sides, which can be calculated from recurring events identified during the cyclic activity of gait. There are numerous methods described in the literature to determine initial contact and toe-off events from the movement of markers attached to the legs (e.g. [17-20]) but it is unlikely that any one of the methods will be applicable to all forms of gait given the diversity of the targeted conditions.

Considering that any recurring event can be used to calculate the delay, we chose the ‘coordination algorithm’ [21] which uses the foremost and rearmost position of the heel marker relative to the sacrum marker (in the direction of progression) as estimates of initial contact (IC) and toe-off (TO) respectively. To detect these gait events, the differences of antero-posterior positions of the left heel to sacrum and right heel to sacrum markers were passed to a 5 Hz low-pass second order Butterworth Filter module. Rising zero crossing of the filtered derivatives (frame-to-frame marker displacements) was detected by monitoring if the actual value was > =0 and the previous value was <0 triggering right and left IC events (RIC and LIC). Detection of falling zero crossing (actual value < =0 and previous value >0) was used to identify right and left TO events (RTO and LTO).

### Delay of mirrored side

**Figure 3:**
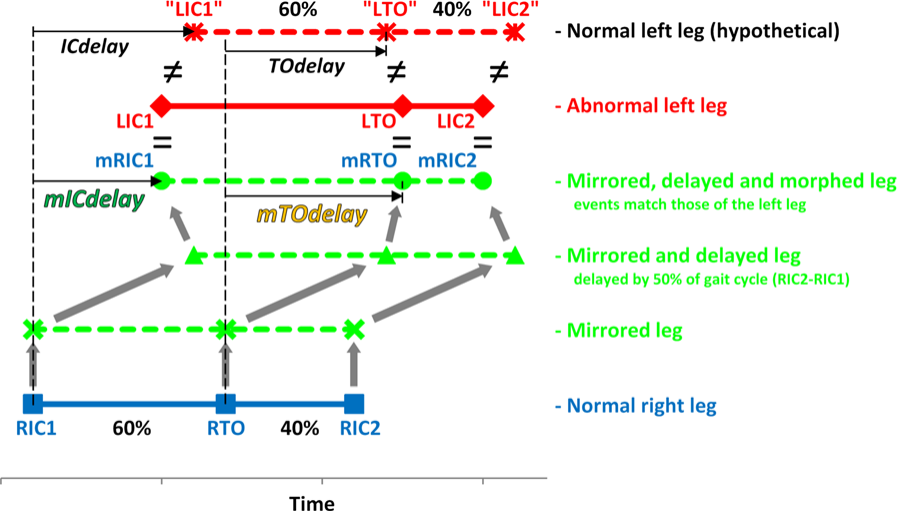
**Gait of the right side can be mirrored and delayed by 50% of the gait cycle.** Temporal morphing is needed however to match the virtual events of the mirrored leg to the physical events of the left side. The two variables of *mICdelay* and *mTOdelay* need to be calculated to describe the delay from one side to the other. (Note that the same logic applies when mirroring the left side.)

In normal gait, TO occurs at approximately 60% of the gait cycle between two consecutive ICs when walking at a preferred speed [22], but in abnormal gait the timing of TO may be altered. In addition, the relative timing of events on the contralateral side may also deviate from normal. Delaying the mirrored side by 50% of the gait cycle would give the patient an illusion of perfectly symmetrical gait but the effect would be tempered by the mismatch between physical and virtual gait events. A feasible compromise is to delay the mirrored side and to temporally “morph” (stretch or compress) the movement between consecutive gait events. In this way the virtual leg can closely follow the reference side’s movement and can also contact and leave the supporting surface at the same time as the physical leg (Figure 3). As the timing of IC and TO on the abnormal side are likely to be different from the hypothetical normal values, the delay of the mirrored IC (*mICdelay*) may be different from the delay of the mirrored TO (*mTOdelay*). For example Figure 3 shows an *mICdelay* of 40% and a *mTOdelay* of 55% as opposed to the hypothetical 50% (both *ICdelay* and *TOdelay*).

**Figure 4:**
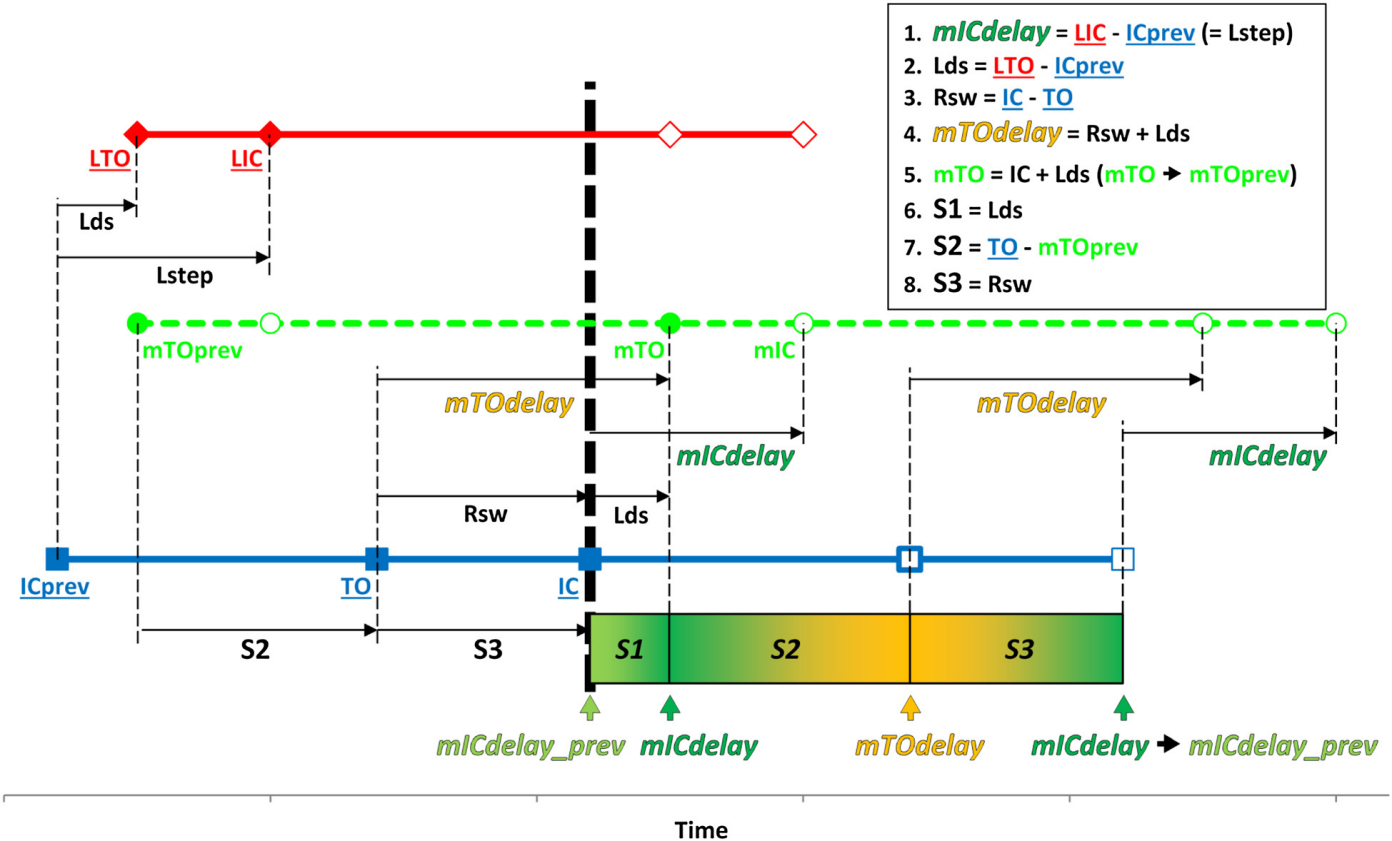
**The timing of events in the mirrored, delayed and morphed left leg (in green, from Figure **3**) are illustrated in relation to gait events of the normal right leg (in blue) and the abnormal left leg (in red).** Two values of delay (*mICdelay* and *mTOdelay*) are calculated at IC (bold vertical dotted line) from IC and TO events of the two sides identified during the previous gait cycle (underlined events). Movement of the avatar’s contralateral side (left side in this case, in red) is then delayed by a smoothly altered value changing from the previous *mICdelay* (*mICdelay_prev*) to *mICdelay* over S1, to *mTOdelay* over S2 and to *mICdelay* over S3. Solid symbols are events used in the calculations, empty symbols indicate the rest of events in the gait cycles. Abbreviations: Lds = Left double support, Rsw = Right swing, also see text.

The absolute values of *mICdelay* and *mTOdelay* can be calculated at the moment of IC from events of the previous gait cycles of the right and left side (Figure 4). To avoid an abrupt change in leg movement when switching between the two different delay values, a sigmoid transfer function was used to change the delay smoothly. The first transition is from the previous *mICdelay* (*mICdelay_prev*) to the current *mICdelay* during a section between IC and mTO (*S1*). The second transition occurs to *mTOdelay* until the next TO (during *S2*), and the third transition returns to *mICdelay* until the next IC (during *S3*) (Figure 4).

### Participant and protocol

Gait of a left sided trans-femoral amputee (age 49 years; height 1.79 m; mass 76 kg; 6 years since amputation) was recorded using the HBM, captured by a 16 camera Vicon MX system while walking at his preferred speed on a treadmill at 2.8 km · h^-1^ for 83 s. The amputee used a KX-06 knee and an Echelon ankle-foot device, both controlled hydraulically (Chas A Blatchford & Sons Ltd, Basingstoke, UK). The VMB software module of the CAREN system mirrored and delayed the right leg and arm markers with reference to mirror planes attached to the pelvis and trunk respectively. Flexion/extension angles of the knees and elbows were re-sampled off-line at 100 Hz with quintic splines to correct for the non-constant sampling rate of the D-Flow software. Cross correlation of angles between the right and left sides was used to quantify lag over a 10 s long clean section (35-45 s) using Matlab 2012b (The Mathworks, Natick MA, USA). Mirroring accuracy was quantified by Root Mean Squared Deviations (RMSD) of the lag-corrected, mirrored and delayed angles of the knees and elbows together with correlation of the corresponding angles. To test the effect of smoothly changing the mirroring delay, IC and TO events of the physical left side and the mirrored and delayed right side were compared with paired t-tests over a section between 24-82 s when gait was in a steady state. All procedures were approved by the institutional ethics committee (11/SPS/037) and the participant provided written informed consent.

## Results

**Figure 5:**
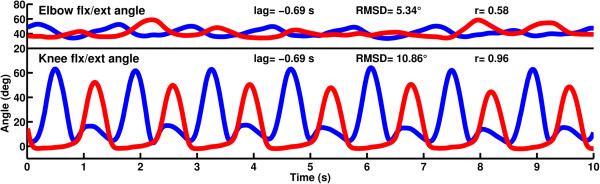
**Elbow flexion/extension and knee flexion/extension angles of the participant over 10 s treadmill walking.** Flexion is positive, extension is negative; right side is in blue, left side is in red.

**Figure 6:**
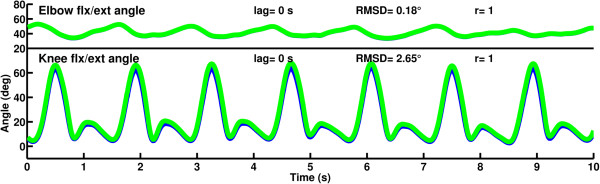
**Elbow and knee flexion/extension angles of the participant while mirroring the right arm and leg in place of the left limbs.** Flexion is positive, extension is negative; right side is in blue, mirrored right side in place of the left side is in green.

The participant’s asymmetrical gait was characterised by a lack of loading response knee flexion on the left side due to the design of the artificial limb, which locks the knee during weight bearing. Overall the left knee was held in a more extended position than the right knee throughout gait (Figure 5). After removing 69 frames of lag (-0.69 s) as derived from cross correlation of the knee angles, the RMSD of angle curves between the knees and elbows was 10.86° and 5.34° respectively. The correlation coefficient between the knee angles was 0.96 and between the elbow angles was 0.58.Mirroring of the right side to the left side without a delay resulted in closely matched angles of the elbow and knee (Figure 6). Cross correlation of the right knee angle and mirrored knee angle showed no lag. The RMSD of the knee flexion/extension angles was 2.65° and the RMSD of the elbow flexion/extension angles was 0.18°. Correlation coefficients between both the knee angles and elbow angles were 1.00.

**Figure 7:**
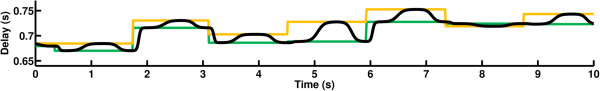
**The bold curve shows the dynamically changing delay during a sequence of several strides.** Values of *mICdelay* and *mTOdelay* are indicated by horizontal green and orange line segments respectively.

The delays of IC (*mICdelay*) and TO (*mTOdelay*) were calculated at every RIC and are indicated by the horizontal lines in Figure 7 for each right gait cycle over 10 s. A smoothly changing delay during each right stride is the result of the continuous morphing. Because the sigmoid transfer function makes the delay approach *mICdelay* and *mTOdelay* asymptotically, any minor deviations of the actual gait events from those in the previous gait cycle do not cause a break in the delay curve.

**Figure 8:**
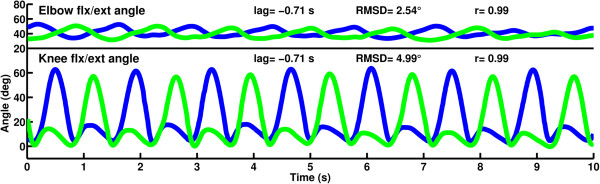
**Almost normal looking bilateral elbow and knee angle profiles result from the mirroring combined with morphed delay.** Flexion is positive, extension is negative; right side is in blue, mirrored right side in place of the left side is in green.

When applying the dynamically calculated morphed delay to the mirrored right side, a normal looking gait pattern is seen on the virtual mirror display (see video, Additional file 1) confirmed by almost symmetrical angle curves of the knees and elbows with a natural phase lag (Figure 8). Note the presence of a normal looking knee loading response flexion and lack of the extension offset on the virtual left side. Cross correlation of the right knee angles and the mirrored and delayed virtual left knee angles showed an offset of 71 samples (-0.71 s). After removing the lag, a RMSD of 4.99° was found between the knee angle curves and 2.54° between the elbow angles. Correlation coefficients between both the knee angles and elbow angles were 0.99.

**Figure 9:**
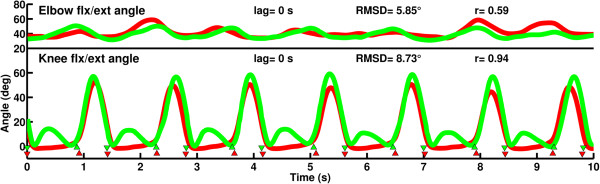
**Angles of the elbow and knee on the patient’s left side (red) and on the avatar’s virtual left side (green).** Gait events are indicated by ▼ (IC) and ▲ (TO) for the physical left side in red and the virtual leg in green.

**Figure 10:**
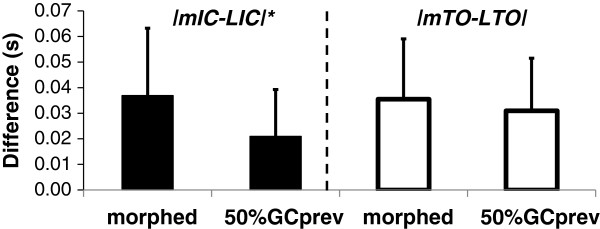
**The differences between virtual and physical events of the left leg (mIC, LIC and mTO, LTO) when applying mirroring combined with morphed delaying, and delaying by 50% of the previous right gait cycle’s duration.** The * indicates a significant difference (p < 0.05).

A comparison of the left knee and elbow angles to the mirrored and delayed angles shows the difference between movement of the impaired left limbs and the virtual limbs (Figure 9). Cross correlation showed no lag and a RMSD of 8.73° between the knees and 5.85° between the elbows. Correlation coefficients were 0.94 and 0.59. Figure 9 also indicates the IC and TO events of the physical and virtual left sides. Over the steady state section of the walking trial (24-82 s covering 41 strides) the difference between the physical and virtual IC events was 0.037 ± 0.026 s and 0.035 ± 0.024 s between the physical and virtual TO events. For reference, the differences when delaying by 50% of the previous right gait cycle duration were 0.021 ± 0.018 s and 0.031 ± 0.021 s for IC and TO respectively (Figure 10). A paired t-test showed that the difference between the physical and virtual IC events was significantly higher when using the morphing method compared to delaying by 50% of the previous gait cycle (t_41_ = 2.01, p = 0.0006). The difference comparing the physical and virtual TO events however was non-significant between the two delaying methods (t_41_ = 2.01, p = 0.21).

## Discussion

The ability of the Virtual Mirror Box to replace the impaired side with virtual limbs was evaluated by comparing movement of an amputee’s impaired limbs to the movement of his virtual limbs generated by the VMB. Specifically the VMB was expected to provide a faithful correction of the movement asymmetry achieved by mirroring and delaying the reference side using a continuously changing adaptive time delay to predict gait events of the mirrored side.

### Effects on the movement of limbs

Visually the virtual leg showed normal movement characteristics taken from the participant’s unaffected leg including normal knee flexion in stance and normal alignment of the whole leg. Asymmetry of the arm movements was also corrected. Using the VMB more than halved the side-to-side RMSD of knee and elbow angles from 10.86° and 5.34° to 4.99° and 2.54° respectively. Correlation coefficients used as measures of symmetry improved from 0.96 and 0.58 to 0.99. The RMSD values found in this study are comparable but higher than our previous findings with normal gait where the RMSD between the original and virtual knees and elbows was 3.5° and 0.9° respectively [23]. This difference may be explained by the unimpaired participant’s more repeatable gait cycles.

Morphing inevitably makes the angles slightly distorted but this is an inevitable compromise for the benefit of temporal congruency between physical and virtual events. For example the RMSD between the physical right leg and virtual left leg is higher after morphing (4.99°) than with mirroring only (without morphing) (2.65°) but in return, the physical and virtual events are expected to match better.

### Effects on gait events of the mirrored side

The application of smooth transitions between *mICdelay* and *mTOdelay* handles asymmetrical gait characterised by an offset and altered proportions of stance and swing phases on the contralateral side. This method however results in a perfect match of virtual and physical events on the mirrored side only if *mICdelay* and *mTOdelay* remain the same over consecutive gait cycles. Gait and especially abnormal gait however exhibits cycle-to-cycle variation in the timing of gait events and so the application of *mICdelay* and *mTOdelay* derived from the previous gait cycles inevitably causes minor discrepancies between the virtual and physical events.

In case of the amputee we tested, the effects of morphing did not reduce the difference between physical and virtual events any better than using 50% of the previous gait cycle as a delay. This can be explained by the cycle-to-cycle variability of gait confirmed by the cycle-to-cycle changes of *mICdelay* and *mTOdelay* (c.f. Figure 7).

Morphing of the delay is preferred over using 50% of the previous gait cycle duration. Firstly, the morphing method causes only a non-significant difference between physical and virtual TO events even if there is cycle-to-cycle variability of gait. The difference between physical and virtual IC events is statistically significant but is only 0.016 s on average, which is only 1.1% of the mean left stride time (1.4 s) and therefore not practically meaningful. Secondly, if the cycle-to-cycle variability was lower, then the difference between the physical and virtual events would be expected to reduce as well as the RMSD between the physical and virtual limbs. If cycle-to-cycle variability of the patient’s gait is available then this can determine if dynamic morphing or 50% of the previous gait cycle duration should be used.

### The roles of mirror planes

In its current implementation of the VMB algorithm, markers of the leg are mirrored to a vertical plane which follows rotation of the pelvis and is attached to the sacrum marker. The hip joint centre on the mirrored side, however, is derived from the pelvis markers which are not mirrored. As a consequence of the hip centre moving together with the pelvis and not the mirrored thigh, the position and orientation of the thigh segment is not an exact mirrored image of the other side when pelvic movements are asymmetrical. This limitation of the mirroring method explains the minor difference found in the knee angle when mirroring, as our participant had considerable asymmetry in pelvic movements. The difference in knee (and hip) angles could be eliminated by mirroring the hip joint but that would cause an apparent dislocation of the mirrored hip joint, which is perhaps more disadvantageous visually than the minor difference in the position and orientation of the thigh and consequential asymmetry of the knee and hip angles.

Contrary to the leg, the arm is mirrored to a trunk-attached mirror plane defined by markers over C7 and T10 spinous processes and the sternum. As a result the mirrored arm markers move together with the shoulder centre better than the leg markers with the hip centre even when there is much lateral trunk sway. Such a definition of the trunk mirror plane also explains why the elbow angles were almost identical when mirroring the arm. A disadvantageous consequence of the trunk mirror plane being attached to the trunk’s local sagittal plane is that with lateral trunk sway the lateral displacement of the arm is amplified. This visual artefact can be eliminated by optionally defining a trunk mirror plane in a vertical plane (similarly to the mirror plane of the pelvis). Such mirroring would however introduce virtual dislocation of the shoulder, similar to the hip joint. Exact effects of using different mirror planes and their impact on visualisation of the avatar should be evaluated in future studies.

### Visual presentation of the virtual avatar

The avatar following movement of the participant manipulated by the VMB algorithm can be presented in a number of ways. A TV screen positioned in front of the treadmill in a portrait orientation replicates the setup commonly used in physiotherapy rooms or fitness centres where mirrors are in front of the treadmills. In addition to the default straight on view, any viewing angle can be shown by rotating, panning and zooming with the virtual camera in D-Flow. A special effect can be achieved by assigning a local coordinate system to a TV display by attaching three reflective markers. The virtual camera in D-Flow can then be linked to the physical display’s position and orientation. A display moved around the participant on wheels can show a changing view as if it was a physical mirror carried around. A head mounted display can also be used to present the manipulated avatar to the participant although the head has to be tilted forwards to see enough of the mirrored leg. Such manipulations of the display can provide numerous options for presenting their movement to participants, some of which may improve the effectiveness of visual feedback [24].

## Conclusions

Thanks to an adaptive time delay calculated continuously from past gait events by the Virtual Mirror Box, for the first time patients with unilateral gait problems can view their own symmetrical gait while walking on a treadmill. A number of inevitable limitations exist due to predicting gait events from variable gait, but overall the VMB can be regarded as a powerful tool for providing visual feedback to patients with a variety of conditions during gait. The clinical effect of such dynamic visual feedback can be evaluated next in amputees, cerebral palsy, stroke and complex regional pain syndrome.

## Competing interests

The authors declare that they have no competing interests. The research grant received by MAR and GJB from LJMU’s Institute for Health Research was used to fund development of the first VMB prototype by Motek Medical, employer of ECPvL and TG.

## Authors’ contributions

GJB conceived of the study, created the final D-Flow application and performed the analysis and interpretation of results. ARDA recruited the patient for testing, participated in the data collection and then in the interpretation of findings. ECPvL developed the initial D-Flow application and TG designed the implementation of the VMB within D-Flow’s MoCap module. MAR participated in conceiving and designing the study. All authors read and approved the final manuscript.

## Supplementary Material

Additional file 1 **Graphical output of the Virtual Mirror Box with adaptive time delay in D-Flow.** The video shows the amputee’s naturally asymmetrical gait, mirrored gait (without delay), and mirrored gait with time delay. The blue and red curves are right and left knee flexion/extension angles, the green and mint curves are right and left elbow flexion/extension curves, respectively. The white curve shows the delay as it changes smoothly between *mICdelay* (yellow) and *mTOdelay* (green) in each gait cycle. Click here for file
